# Impact of robotic-assisted versus video-assisted thoracoscopic surgery on efficacy and quality of life in patients with pulmonary opacities: a propensity score-matched analysis

**DOI:** 10.3389/fonc.2025.1598990

**Published:** 2025-07-25

**Authors:** Liuchun Huang, Shengjing Liang, Jing He, Junqi Qin, Jiaping Wei, Jianwei Huang, Shucong Peng, Xianglan Chen, Chunhui Pan, Huifang Pen, Yu Wen, Yifan Zhou, Yonglong Zhong

**Affiliations:** Department of Thoracic Surgery, The People’s Hospital of Guangxi Zhuang Autonomous Region, Guangxi Academy of Medical Sciences, Nanning, China

**Keywords:** robotic-assisted thoracoscopic surgery, video-assisted thoracoscopic surgery, pulmonary opacities, perioperative outcomes, postoperative quality of life

## Abstract

**Objective:**

With the increasing detection of pulmonary opacities through low-dose computed tomography (LDCT), minimally invasive surgical techniques have gained prominence. While debates persist regarding the comparative efficacy and postoperative quality of life (QOL) between robot-assisted thoracoscopic surgery (RATS) and video-assisted thoracoscopic surgery (VATS), this study aimed to compare the perioperative outcomes and postoperative QOL between RATS and VATS in patients with pulmonary opacities.

**Methods:**

A retrospective analysis was performed on patients who underwent pulmonary opacities resection at the Department of Thoracic Surgery, Guangxi Zhuang Autonomous Region People’s Hospital, between January 2021 and November 2024. Patient characteristics, perioperative clinical indicators, economic parameters, and QOL scores were analyzed.

**Results:**

A total of 1,173 patients undergoing RATS and VATS was conducted, after propensity score matching, 277 pairs of patients entered into final analysis. Baseline characteristics were similar between groups. Compared with VATS, RATS had shorter operative time (145.35 ± 48.51 vs. 170.39 ± 71.49, *p* < 0.001), less intraoperative blood loss [20 (10, 20) mL vs. 30 (20, 100) mL, *p* < 0.001], shorter chest tube duration (3.82 ± 1.28 vs. 4.28 ± 2.63 days), less 48-hour postoperative drainage (217.73 ± 107.69 mL vs. 244.01 ± 120.48 mL, *p* = 0.007), shorter postoperative hospital stay (6.05 ± 1.54 vs. 7.14 ± 5.04, *p* = 0.001), and lower overall postoperative complication rate than the VATS group (9.39% vs. 15.52%, *p* = 0.029). But, total hospitalization costs were higher in the RATS group. Moreover, the Visual Analogue Scale (VAS) score on postoperative day 3 in the RATS group was significantly lower (*p* < 0.001). At 3 months postoperatively, the RATS group reported significantly less pain/discomfort (*p* = 0.015) in the QOL assessment.

**Conclusion:**

RATS indicated shorter operative time, reduced postoperative complications, shorter hospital stay, less intraoperative blood loss and less compared to VATS in patients with pulmonary opacities. Although hospitalization costs were higher, RATS was associated with improved postoperative pain management and QOL regarding pain/discomfort.

## Introduction

With the widespread application of low-dose computed tomography (LDCT) in lung cancer screening, the detection rate of pulmonary opacities has increased significantly (1). Globally, approximately 10%-20% of adults are incidentally found to have pulmonary opacities on imaging studies, some of which may represent early-stage lung cancer or precancerous lesions (2). Surgical resection remains a critical approach for the diagnosis and treatment of pulmonary opacities, and technological advancements in this field continue to be a research focus in thoracic surgery. Traditional open thoracotomy, characterized by substantial trauma and prolonged postoperative recovery (3), has gradually been replaced by minimally invasive techniques such as video-assisted thoracoscopic surgery (VATS) (4). However, VATS also presents technical limitations, including the loss of depth perception due to its two-dimensional visual field and restricted maneuverability of straight-shaft instruments (5).

In recent years, the introduction of the Da Vinci Surgical System has further advanced the precision and intelligence of thoracic surgical procedures (6). More and more clinical centers are using robotic systems to perform thoracic surgery, including lung cancer. Nevertheless, there remains ongoing debate regarding the differences in therapeutic efficacy and postoperative quality of life between the two minimally invasive approaches—robot-assisted thoracoscopic surgery (RATS) and VATS—for patients with pulmonary opacities. This study aims to analyze the perioperative outcomes and postoperative quality of life between RATS and VATS in patients with pulmonary opacities through a retrospective analysis, thereby providing evidence to support individualized clinical decision-making.

## Methods

### Study design and general information

This prospective, observational study included 1173 patients with pulmonary opacities was performed at the Department of Thoracic Surgery of People’s Hospital of Guangxi Zhuang Autonomous Region between January 2021 and November 2024. The location of the lung opacities included both central and peripheral types (central type: 2 cm from the hilum of the lung; peripheral type: sub-pleural or within the outer one-third of the lung). Systematic collection and analysis of clinical data were conducted to assess postoperative effects and short-term quality of life.

The inclusion criteria were as follows: (1) consent for RATS or VATS segmentectomy/lobectomy, (2) no preoperative radiotherapy, chemotherapy, or other treatments, (3) not combined with severe heart, lung, liver, and kidney function damage. The exclusion criteria were as follows: (1) limited activity or psychiatric disorders, (2) loss to follow-up, (3) wedge resection of lung, as a definitive treatment. (4) pathological staging missing.

Participants were divided into two groups: the RATS group and the VATS group. Participants self-selected either RATS or VATS based on financial situation and were assigned to the respective groups accordingly. Perioperative clinical parameters were analyzed between the two groups, including: age, gender, Body Mass Index (BMI), smoking history, tumor location, tumor size (based on preoperative CT measurements), surgical approach, type of lymphadenectomy, operative time, whether conversion to open surgery or not, pathological type, lymph node status, postoperative pathological stage [based on 8^th^ edition of the tumor, node, and metastasis (TNM) classification of lung cancer], postoperative complications, distribution of complications, intraoperative blood loss, chest tube duration, postoperative 24h drainage, postoperative 48h drainage, postoperative hospital stay, total cost for hospital stay, postoperative pain scores on day 1 and day 3 (visual analog scale, VAS), and health-related quality of life score (EQ-5D-3L). This study was approved by the Ethics Committee of Guangxi Zhuang Autonomous Region People’s Hospital (KY-ZC-2023-080), and written informed consent was obtained from all participants.

### Anesthesia and surgical procedures

#### RATS group

General anesthesia was induced using a combined intravenous and inhalation approach, with double-lumen endotracheal intubation to ensure single-lung ventilation. Patients were positioned in the lateral decubitus position, and three robotic arms were deployed. The camera port (8 mm robotic trocar) was placed at the 8th or 9th intercostal space (ICS) along the mid-axillary line. Auxiliary ports were positioned at the 10th ICS (posterior axillary line) and the 8th ICS (anterior axillary line), while the main operating port was established at the 4th or 5th ICS (anterior axillary line). Robotic-specific instruments (fenestrated bipolar forceps and permanent cautery look) and a three-dimensional endoscope were introduced through these ports. If the surgery involved suturing operations, a large needle driver would be used. Considering the issue of cost, the robotic linear cutting stapler was not used in the RATS operation. After layered closure of the thoracic cavity, a chest tube was inserted for drainage.

#### VATS group

Patients were anaesthetized and positioned similarly to the RATS group. A uniportal or biportal VATS approach was employed. For uniportal VATS, a single incision (2 cm - 4 cm in length) was created at the 4th or 5th ICS along the anterior axillary line, serving as the main operating port. In biportal VATS, an additional observation port (1 cm incision) was placed at the 6th or 7th ICS along the mid-axillary line for thoracoscope insertion. Surgical procedures, including anatomical dissection, lymph node management, and chest tube placement, were identical to those in the RATS group, with the exception of robotic platform utilization.

### Surgical method selection

Carbon dioxide insufflation was not employed in either RATS or VATS group, and the natural pneumothorax condition was maintained throughout the procedure. The type of anatomical resection was rigorously based on oncological principles and guideline consensus. Target pulmonary arteries, veins, and bronchi were meticulously dissected and identified based on preoperative CT or three-dimensional reconstruction imaging. For high-risk peripheral pulmonary opacities, the step-by-step mode of “wedge resection → secondary anatomical resection” was used. Lobectomy was preferred for pulmonary opacities larger than 2 cm or high-risk opacities with consolidation tumor ratio (CTR) > 0.5. Segmentectomy was preferred for peripheral nodules less than 2 cm and CTR ≤ 0.5. In addition, segmentectomy was also considered in elderly patients, insufficient lung function reserve, multi-primary cancer needs to retain lung parenchyma or specific inert subtypes, and it needed to be combined with multidisciplinary assessment (MDT) and the patient’s wishes. Before the procedure, CT-guided percutaneous pulmonary nodule puncture and localization using a metal hook was performed for deep lesions that were not easily palpable or small lesions (≤1 cm or > 5 mm from the pleura) as well as pure ground-glass nodules. The modified inflation-deflation was used to define the inter-segment and sub-segment interface of the lungs. Lymph node dissection was guided by intraoperative frozen section pathology report of lung opacity. Lymph node sampling was performed for adenocarcinoma *in situ* or minimally invasive adenocarcinoma, whereas systematic or lobe-specific lymph node dissection was conducted for invasive adenocarcinoma.

### Postoperative management

All patients were managed under a standardized enhanced recovery after surgery (ERAS) protocol. Postoperative rehabilitation exercises were supervised by a dedicated pulmonary rehabilitation therapist, with urinary catheter removal and early ambulation initiated within 24–48 hours postoperatively. Laxatives were administered as needed, and pain was controlled using oral/intravenous analgesics. Postoperative pain score was systematically evaluated by independent researchers.

The criteria for chest tube removal included: (1) radiographic confirmation of adequate pulmonary re-expansion; (2) absence of air leakage; and (3) drainage volume < 200 mL over 24 hours. Prolonged air leakage was defined as air leakage exceeding 5 days after surgery. After being discharged from the hospital, the patient would return to the hospital for monthly outpatient review or receive structured telephone follow-up.

### Quality of life assessment using EQ-5D-3L

Health-related quality of life (HRQoL) was evaluated using the EuroQol 5-Dimension 3-Level (EQ-5D-3L) questionnaire, a standardized and validated instrument for measuring generic health status (7). The EQ-5D-3L included five dimensions: mobility, self-care, usual activities, pain/discomfort, and anxiety/depression, each rated on a three-level severity scale (no problems, moderate problems, or extreme problems). Additionally, a visual analog scale (VAS) ranging from 0 (“worst imaginable health state”) to 100 (“best imaginable health state”) was included to capture patients’ self-rated overall health status. The utility index derived from the EQ-5D-3L was calculated using population-specific value sets to reflect health state preferences. Data were collected at predefined intervals to ensure temporal consistency and minimize recall bias.

### Data analysis

To mitigate the impact of nonrandom patient allocation and confounding variables, a propensity score matching (PSM) analysis was conducted. Balanced variables for matching included patient age, BMI, gender, smoking history, tumor location, tumor size, surgical approach, and type of lymphadenectomy. This process utilized the optimal algorithm with a 1:1 matching ratio and a caliper value set at 0.02.

All statistical analyses were performed using IBM SPSS Statistics version 26.0. For quantitative data, the distribution shape and homogeneity of variances were verified. Normally distributed data were analyzed using the t-test and are presented as means ± standard deviations (mean ± SD). If the data did not follow a normal distribution, medians (interquartile ranges) [M(IQR)] were used, and comparisons were made using the Mann-Whitney U test. Categorical data were analyzed using the chi-square test or fisher test. A P-value of less than 0.05 was considered statistically significant.

## Results

### Patient enrollment and baseline characteristics

From January 2021 and November 2024, a total of 1,173 patients were included in the final analysis. Among them, 290 patients underwent RATS and 883 patients received VATS. There was no recorded mortality within either the 30-day or 90-day post-treatment periods in this study. As shown in **Table 1**, after PSM, 277 pairs (554 patients) were created in this study. No significant differences were observed between the two groups in baseline characteristics, including age, BMI, gender, smoking history, tumor location, tumor size, surgical approach and type of lymphadenectomy (all *p* > 0.05).

**Table 1:** Demographic and clinical characteristics of patients with pulmonary opacity.

Variables	Before matching				After matching			
	RATS group (n=290)	VATS group (n=883)	t/χ²	P-value	RATS group (n=277)	VATS group (n=277)	t/χ²	P-value
Age, years, mean (SD)	59.23 ± 10.875	59.15 ± 191.02	0.111	0.911	59.58 ± 10.756	58.99 ± 12.10	0.612	0.540
BMI, kg/m²	24.10 ± 16.86	23.38 ± 6.91	1.037	0.300	23.15 ± 3.02	24.07 ± 11.33	-1.315	0.189
Gender			0.374	0.541				
male	129 (44.48%)	411 (46.55%)			118 (42.60%)	118 (42.60%)	0.000	1.000
female	161 (55.52%)	472 (53.45%)			159 (57.40%)	159 (57.40%)		
Smoking history			14.019	<0.001*			0.029	0.865
Yes	19 (6.55%)	133 (15.06%)			18 (6.50%)	19 (6.86%)		
No	271(93.45%)	750 (84.94%)			259 (93.50%)	258 (93.14%)		
Tumor location			7.057	0.133			1.992	0.737
Right upper lobe	97 (33.45%)	238 (26.95%)			88 (31.77%)	85 (30.69%)		
Right middle lobe	22 (7.59%)	103 (11.66%)			21 (7.58%)	19 (6.86%)		
Right lower lobe	63 (21.72%)	202 (22.88%)			61 (22.02%)	75 (27.08%)		
Left upper lobe	64 (22.07%)	193 (21.86%)			64 (23.10%)	59 (21.30%)		
Left lower lobe	44 (15.17%)	147 (16.65%)			43 (15.52%)	59 (21.30%)		
Tumor size (T), cm			5.173	0.160			3.981	0.264
T ≤ 3.0	246 (84.83%)	718 (81.31%)			234 (88.48%)	244 (88.09%)		
3.0<T ≤ 5.0	34 (11.72%)	103 (11.66%)			33 (11.91%)	22 (7.94%)		
5.0<T ≤ 7.0	8 (2.76%)	43 (4.87%)			8 (2.89%)	6 (2.17%)		
T>7.0	2 (0.39%)	19 (2.15%)			2 (0.72%)	5 (1.81%)		
Surgical approach			46.936	<0.001*			0.065	0.799
Segmentectomy	151 (52.07%)	264 (29.90%)			139 (50.18%)	136 (49.10%)		
Lobectomy	139 (47.93%)	619 (70.10%)			138 (49.82%)	141 (50.90%)		
Type of lymphadenectomy			2.231	0.135			0.174	0.676
Systematic dissection	276 (95.17%)	818 (92.64%)			264 (95.31%)	266 (96.03%)		
Selective sampling	14 (4.83%)	65 (7.36%)			13 (4.69%)	11 (3.97%)		

BMI, Body Mass Index.

RATS, robot-assisted thoracoscopic surgery; VATS, video-assisted thoracoscopic surgery.

*p value less than 0.05.

### Comparative analysis of perioperative clinical and economic indicators

**Table 2** presents the comparison of clinical observation indicators and economic parameters between RATS and VATS groups. The VATS group required a mean operative time of 170.39 ± 71.49 minutes, whereas the robotic group completed procedures in 145.35 ± 48.51 minutes, representing a 25-minute reduction averagely in the robotic cohort (*p* < 0.001). The proportion of intraoperative conversion to open surgery in the two groups was similar, and there was no statistical difference.

**Table 2:** Conventional clinical characteristics of patients undergoing pulmonary opacity resection.

Variables	RATS group (n=277)	VATS group (n=277)	t/χ²/Z value	*P*-value
Operative time (min)	145.35 ± 48.51	170.39 ± 71.49	-4.824	<0.001*
Conversion to open surgery			0.202	0.653
Yes	2 (0.7%)	3 (1.1%)		
No	275 (99.3%)	274 (98.9%)		
Pathological type			0.000	1.000
Adenocarcinoma	229 (82.67%)	229 (82.67%)		
Non-adenocarcinoma	48 (17.33%)	48 (17.33%)		
Lymph node status			0.218	0.641
Positive	24 (8.66%)	21 (7.58%)		
Negative	253 (91.34%)	256 (92.42%)		
Postoperative pathological stage			0.283	0.595
Stage I	243 (87.7%)	247 (89.2%)		
Stage II-III	34 (12.3%)	30 (10.8%)		
Postoperative complications			4.784	0.029*
Yes	26 (9.39%)	43 (15.52%)		
None	251 (90.61%)	234 (84.48%)		
Distribution of complications			6.248	0.100
Prolonged air leak	14 (5.05%)	24 (8.66%)		
Pneumonia	7 (2.53%)	15 (5.42%)		
Others	5 (1.81%)	4 (1.44%)		
None	251 (90.61%)	234 (84.48%)		
Intraoperative blood loss (mL)	20 (10, 20)	30 (20, 100)	-6.755	<0.001*
Chest tube duration, days (range)	3.82 ± 1.28	4.28 ± 2.63	2.65	0.008*
Postoperative 24h drainage (mL)	192.80 ± 198.78	201.66 ± 151.84	0.59	0.556
Postoperative 48h drainage (mL)	217.73 ± 107.69	244.01 ± 120.48	2.71	0.007*
Postoperative hospital stay, days	6.05 ± 1.54	7.14 ± 5.04	3.46	0.001*
Total cost for hospital stay, CNY	67236.22 ± 11341.44	47398.22 ± 13073.69	19.08	<0.001*
Pain score at POD 1	4.92 ± 2.00	5.23 ± 1.98	1.88	0.064
Pain score at POD 3	3.23 ± 1.29	4.17 ± 1.79	7.12	<0.001*

Others including Loculated pleural effusion, Chylothorax, Cerebral Infarction.

CNY, Chinese Yuan.

POD, Postoperative Day.

Postoperative pathological analysis revealed comparable patterns between groups, with pulmonary adenocarcinoma constituting the predominant diagnosis (over 82% in both groups). Non-adenocarcinoma pathologies (including atypical adenomatous hyperplasia, adenocarcinoma *in situ*, squamous cell carcinoma, and large cell carcinoma) showed similar low incidence rates without intergroup difference. Moreover, there was no difference in postoperative lymph node status and pathological stage in RATS and VATS group.

The RATS group exhibited significantly lower postoperative complication rates (9.39% vs. 15.52%, *p* =0.029). Both groups revealed comparable complication profiles, with prolonged air leak and pneumonia being most prevalent. Less frequent complications (pulmonary embolism, cerebral infarction, chylothorax, and wound infection) showed similar low incidence rates between the RATS and VATS groups.

Compared with VATS, RATS had less intraoperative blood loss [20 (10, 20) mL vs. 30 (20, 100) mL, *p <*0.001], shorter chest tube duration (3.82 ± 1.28 days vs. 4.28 ± 2.63 days), less 48-hour postoperative drainage (217.73 ± 107.69 mL vs. 244.01 ± 120.48 mL, *p* = 0.007), and shorter postoperative hospital stay (6.05 ± 1.54 mL vs. 7.14 ± 5.04 mL, *p* = 0.001). No significant differences were found in 24-hour postoperative drainage (*p* = 0.556). Postoperative pain assessment was analyzed in this study. The VAS scores of the RATS group on postoperative day1 was lower than that of the VATS group, but there was no statistical difference. While, the RATS group had significantly lower VAS scores on postoperative day 3 compared to the VATS group (3.23 ± 1.29 vs. 4.17 ± 1.79, *p* < 0.001). Moreover, total hospitalization costs were significantly higher in the RATS group than that in VATS group (CNY: 67236.22 ± 11341.44 vs. 47398.22 ± 13073.69, *p* < 0.001).

### Comparison of 3-month postoperative quality of life scores (EQ-5D-3L)

In this study, the EQ-5D-3L questionnaire was used to compare perioperative quality of life differences between the RATS and VATS groups. As shown in **Table 3**, there were no significant preoperative differences between the two groups across the five dimensions: mobility, self-care, usual activities, pain/discomfort, and anxiety/depression, confirming baseline comparability.

**Table 3:** Preoperative and 3-month postoperative EQ-5D-3L quality of life scores.

Dimension	Preoperative				Postoperative			
	RATS group, n (%)	VATS group, n (%)	χ²	P-value	RATS group, n (%)	VATS group, n (%)	χ²	P-value
Mobility			3.029	0.220			1.392	0.499
Level 1	272 (98.2%)	276 (99.6%)			253 (91.3%)	247 (89.2%)		
Level 2	3 (1.1%)	10 (0.4%)			23 (8.3%)	27 (9.7%)		
Level 3	2 (0.7%)	0 (0.0%)			1 (0.4%)	3 (1.1%)		
Self-care			4.119	0.128			3.324	0.90
Level 1	2773 (98.6%)	265 (95.7%)			263 (94.9%)	255 (92.1%)		
Level 2	3 (1.1%)	9 (3.2%)			13 (4.7%)	17 (6.1%)		
Level 3	1 (0.4%)	3 (1.1%)			1 (0.4%)	5 (1.8%)		
Usual activities			5.056	0.080			0.308	0.857
Level 1	273 (98.6%)	264 (95.3%)			248 (89.5%)	244 (88.1%)		
Level 2	3 (1.1%)	11 (4.0%)			27 (9.7%)	31 (11.2%)		
Level 3	1 (0.4%)	2 (0.7%)			2 (0.7%)	2 (0.7%)		
Pain/discomfort			/	/			8.350	0.015*
Level 1	277 (100.0%)	277 (100.0%)			213 (78.3%)	184 (68.9%)		
Level 2	/	/			52 (19.5%)	79 (29.6%)		
Level 3	/	/			2 (0.7%)	4 (1.5%)		
Anxiety/depression			1.832	0.400			5.595	0.061
Level 1	272 (98.2%)	267 (96.4%)			255 (92.1%)	241(87.0%)		
Level 2	3 (1.1%)	5 (1.8%)			22 (7.9%)	33 (11.9%)		
Level 3	2 (0.7%)	5 (1.8%)			0 (0.0%)	3 (1.1%)		

EQ-5D-3L, EuroQol Five-Dimensional Three-Level Questionnaire.

RATS, robot-assisted thoracoscopic surgery; VATS, video-assisted thoracoscopic surgery.

*p value less than 0.05.

At 3 months postoperatively, the RATS group exhibited slightly higher proportions of level 1 (no problems) in mobility and self-care compared to the VATS group, while showing marginally lower proportions of level 1 in usual activities and anxiety/depression. However, none of these differences reached statistical significance (*p* > 0.05). Notably, in the postoperative pain/discomfort dimension, the RATS group showed a significantly higher proportion of level 1 (no pain/discomfort: RATS: 78.3% vs. VATS: 68.9%), with correspondingly lower proportions of level 2 (moderate: 19.5% vs. 29.6%) and level 3 (severe: 0.7% vs. 1.5%) (*p* = 0.015).

## Discussion

The widespread application of LDCT has significantly improved the detection rate of pulmonary opacities, thereby driving the rapid development of minimally invasive surgical techniques (8). Traditional VATS, characterized by minimal trauma and rapid postoperative recovery, has become the mainstream approach for pulmonary nodule or mass treatment. In recent years, the da Vinci RATS, leveraging the flexibility of robotic arms and three-dimensional high-definition visualization, has further enhanced surgical precision and gained popularity in numerous centers, earning recognition among thoracic surgeons (9–12). However, debates persist regarding whether the da Vinci robotic system offers substantial clinical advantages.

This study analyzed perioperative outcomes and postoperative QOL between RATS and VATS in patients with pulmonary opacities. In this study, a retrospective analysis of 1,173 patients was conducted, after propensity score matching, 277 pairs of patients entered into final analysis. several critical findings have been drawn: the RATS group showed a significantly shorter operative time, less intraoperative blood loss, shorter chest tube duration, less postoperative drainage, and shorter postoperative hospital stay. These findings were similar to previous small sample size studies (13–16).

Haruki et al. (17) found that the median overall operative time of robotic surgery was reduced by 29 min in Japan. Consistent with this, our results revealed that the operative time in the RATS group was approximately 25 minutes shorter than that in the VATS group (145.35 minutes vs. 170.39 minutes). Conventional VATS, constrained by its two-dimensional visualization and limited instrument maneuverability, may compromise surgical efficiency in complex anatomical regions (e.g., hilar structures or deep-seated opacities (18) due to inadequate depth perception. In contrast, the da Vinci system’s three-dimensional high-definition imaging, 7-degree-of-freedom articulating instruments, and tremor filtration significantly enhance operative precision within the confined thoracic cavity, particularly in technically demanding procedures such as segmentectomies (19–21). For instance, during intersegmental plane dissection, robotic instruments enable more precise differentiation between pulmonary parenchyma and vascular branches, thereby minimizing inadvertent injury (18). Furthermore, the robotic “master-slave” operative configuration allows surgeons to perform procedures in an ergonomic posture, potentially shortening the learning curve and mitigating fatigue-related impacts on surgical efficiency (22–24).

Notably, the reduced operative time in RATS did not compromise safety of surgery. The RATS group exhibited significantly lower intraoperative blood loss compared to the VATS group (mean: 44.68 mL vs. 90.90 mL), suggesting superior stability in vascular management (e.g., pulmonary vessel dissection and ligation). Previous studies have posited that the precise movements of robotic instruments reduce tissue traction injuries (19), a hypothesis further validated by our large-sample data. Additionally, reduced intraoperative bleeding may correlate with diminished postoperative drainage (lower 48-hour drainage volume in RATS group) and shorter chest tube duration, offering insights for future investigations into recovery mechanisms.

Moreover, previous studies have found that RATS have more advantages in lymph node anatomy. For example, the RATS group achieved a more thorough lymph node dissection, with both the number of lymph nodes dissected and the number of stations dissected being greater than those in the VATS group (13, 25). Therefore, in this study, the type of lymph node resection was included as a matching variable without statistical analysis.

Postoperative complications are an important indicator of the safety and effectiveness of surgery. In terms of postoperative complications, Haruki et al. (15) found that the incidence of all complications in the RATS group was not significantly different from that of VATS group; however, the incidence of postoperative pneumonia was significantly lower than that of the VATS group. In this study, the results suggested that the RATS group indicated a significantly lower overall postoperative complication rate than the VATS group (9.39% vs. 15.52%, *p* = 0.029), with similar distributions of complication types (e.g., pneumonia, prolonged air leak). While conventional VATS relies on triangulation of instruments through uniportal, biportal or multiple ports creates torsional forces on intercostal nerves, robotic approach utilized parallel instrumentation via a single incision. The wristed instruments pivot within the thoracic cavity rather than at the chest wall, minimizing mechanical traction on neurovascular bundles. Moreover, the reasons for the more minimally invasive robot operation may be as follows, firstly, robotic precision minimizes non-target tissue damage, such as improved identification of vagus nerves or thoracic ducts during lymphadenectomy (26). Secondly, enhanced visualization of microvascular structures under three-dimensional imaging may lower postoperative hemorrhage risks (15). Finally, reduced intraoperative manipulation of pulmonary parenchyma decreases alveolar trauma, potentially mitigating reactive pleural effusion (16). Although this study did not analyze the pathophysiological mechanisms underlying specific complications, these hypotheses align with existing literature on reduced tissue trauma in robotic surgery.

Numerous studies have focused on comparisons of traditional clinical outcomes, while others have examined changes in postoperative quality of life (QOL). However, the majority of these investigations have been predominantly focused on single aspects such as pain or cough in QOL assessments (27–29). Wang et al. (27–29) found that, for patients with early-stage non-small-cell lung cancer, compared to the VATS group, the patients in the RATS group experienced less pain, with lower total drug dose of intramuscular diclofenac sodium lidocaine injection and oral celecoxib capsules and reduced pain scores. Similarly, in this study, RATS group had superior postoperative pain management (as evidenced by lower VAS scores at POD 3).

Postoperative psychological rehabilitation and functional recovery are issues of increasing concern to patients, which have been shown to have a positive effect on the postoperative recovery of lung cancer patients (30). In this study, the EQ-5D-3L questionnaire was used to evaluate postoperative functions of patients. This tool has demonstrated strong reliability and sensitivity in surgical populations, making it appropriate for comparing postoperative recovery outcomes between intervention groups. In postoperative QOL assessments of this study, the robotic surgery indicated comparable quality of life scores to thoracoscopic surgery in the dimensions of Mobility, Self-Care, Usual Activities, and Anxiety/Depression. As shown in **Table 3**, although both groups exhibited comparable performance across multiple dimensions, patients in the RATS group reported significantly less pain/discomfort. In detail, the RATS group’s advantage in the “pain/discomfort” domain (level 1: 78.3% vs. 68.9%; level 2: 19.5% vs. 29.6%; level 3: 0.7% vs. 1.5%) warrants attention. These findings suggested that while both groups experienced postoperative incisional pain, the RATS group reported significantly less pain intensity compared to the VATS group followed up 3 months after surgery. Despite both groups undergoing minimally invasive procedures, the improved pain perception in RATS may stem from: Smaller-diameter robotic instruments (0.8 cm) and ergonomic designs reducing intercostal nerve compression; Diminished tissue injury lowering inflammatory mediator release; Shorter operative time alleviating sustained thoracic wall pressure (31). While no significant difference was observed in the “anxiety/depression” domain, improved pain control may indirectly influence psychological status by reducing analgesic (e.g., opioid) usage—a hypothesis requiring prospective validation.

Despite RATS’s clinical advantages, this study also identified a nearly 30% increase in total hospitalization costs for the RATS group (CNY: 67236.22 ± 11341.44 vs. 47398.22 ± 13073.69, *p* < 0.001), primarily attributable to specialized consumables (fenestrated bipolar forceps and permanent cautery look). Wang et al. (14) reported that the da Vinci robot costs about $2900 USD once and is not covered by most commercial and government insurance. The high acquisition and maintenance costs of the da Vinci system remain a barrier to its widespread adoption (32, 33). However, focusing solely on direct medical costs may underestimate RATS’s long-term benefits. For instance, the 7.45% reduction in postoperative complications could lower subsequent treatment expenses, while faster recovery may reduce societal costs via earlier return to work. Moreover, enhanced precision may decrease reoperation rates. Nevertheless, cost disadvantages may diminish with future advancements in medical materials (34).

Admittedly, this study has several limitations. First, its single-center, prospective, observational design may introduce confounding variables, such as surgeon learning curves or selection bias (e.g., preferential use of robotics for complex cases). Second, the omission of pulmonary function recovery or long-term survival data in QOL assessments may underestimate RATS’s extended benefits (35). Third, the economic analysis focused solely on hospitalization costs, necessitating comprehensive cost-effectiveness models. Fourth, this study has not yet compared the postoperative pulmonary function. The analysis of follow-up data on lung function for 6 months and 12 months after surgery is still being collected and collated. Fifth, this study includes solid nodules and sub-solid nodules (including s pure ground-glass opacities and part-solid nodules with some solid components), and its classification strictly complies with the imaging standards set by the Fleischner Society. Due to limited research time, it is difficult to systematically quantify the specific distribution ratio of these two types of nodules and the proportion of solid components. Finally, due to the limited research time, the data on preoperative comorbidities (Diabetes, hypertension, coronary heart disease, hyperlipidemia, chronic obstructive pulmonary disease, stroke, etc.) have not been collected statistically, which may affect the interpretation of the research conclusions. In addition, systematically reclassifying all complications according to the Clavien-Dindo scale and precisely separating them into intraoperative vs. postoperative categories for tabulation is not feasible within the available timeframe. Future research will involve multicenter randomized controlled trials to track patient QOL and oncologic outcomes beyond one year postoperatively, further evaluating RATS’s long-term cost-effectiveness.

## Conclusion

This study show that RATS offers distinct advantages over VATS in the treatment of pulmonary opacities, including shorter operative time, reduced intraoperative blood loss, lower postoperative complication rates, and improved pain management. The RATS group also exhibited superior quality of life outcomes at 3 months postoperatively, particularly in pain/discomfort domains. However, the higher total hospitalization costs associated with RATS necessitate careful consideration of cost-effectiveness in clinical decision-making. Limitations of this study include its retrospective design and potential selection bias. Future prospective, multicenter studies with long-term follow-up are warranted to validate these results and evaluate the economic impact of RATS adoption.

## Data Availability

The original contributions presented in the study are included in the article/supplementary material. Further inquiries can be directed to the corresponding authors.

## References

[1] Hendrick RE Smith RA. Benefit-to-radiation-risk of low-dose computed tomography lung cancer screening. Cancer-Am Cancer Soc. (2024) 130:216–23. doi: 10.1002/cncr.34855, PMID: 37909872

[2] Zheng S Shu J Xue J Ying C. CT signs and differential diagnosis of peripheral lung cancer and inflammatory pseudotumor: A meta-analysis. J Healthc Eng. (2022) 2022:3547070. doi: 10.1155/2022/3547070, PMID: 35028118 PMC8749376

[3] Nguyen DM Sarkaria IS Song C Reddy RM Villamizar N Herrera LJ. Clinical and economic comparative effectiveness of robotic-assisted, video-assisted thoracoscopic, and open lobectomy. J Thorac Dis. (2020) 12:296–306. doi: 10.21037/jtd.2020.01.40, PMID: 32274096 PMC7139048

[4] Ye B Wang M. Video-assisted thoracoscopic surgery versus thoracotomy for non-small cell lung cancer: A meta-analysis. Comb Chem High Throughput Screen. (2019) 22:187–93. doi: 10.2174/1386207322666190415103030, PMID: 30987564

[5] Pischik VG. Technical difficulties and extending the indications for VATS lobectomy. J Thorac Dis. (2014) 6:S623–30. doi: 10.3978/j.issn.2072-1439.2014.10.11, PMID: 25379200 PMC4221338

[6] Veronesi G. Robotic surgery for the treatment of early-stage lung cancer. Curr Opin Oncol. (2013) 25:107–14. doi: 10.1097/CCO.0b013e32835daf4f, PMID: 23302938

[7] Khan I Morris S Pashayan N Matata B Bashir Z Maguirre J. Comparing the mapping between EQ-5D-5L, EQ-5D-3L and the EORTC-QLQ-C30 in non-small cell lung cancer patients. Health Qual Life Outcomes. (2016) 14:60. doi: 10.1186/s12955-016-0455-1, PMID: 27072351 PMC4830017

[8] Bonney A Malouf R Marchal C Manners D Fong KM Marshall HM. Impact of low-dose computed tomography (LDCT) screening on lung cancer-related mortality. Cochrane Database Syst Rev. (2022) 8:D13829. doi: 10.1002/14651858.CD013829.pub2, PMID: 35921047 PMC9347663

[9] Shagabayeva L Fu B Panda N Potter AL Auchincloss HG Mansur A. Open, video- and robot-assisted thoracoscopic lobectomy for stage II-IIIA non-small cell lung cancer. Ann Thorac Surg. (2023) 115:184–90. doi: 10.1016/j.athoracsur.2022.01.026, PMID: 35149049

[10] Jin R Zheng Y Yuan Y Han D Cao Y Zhang Y. Robotic-assisted versus video-assisted thoracoscopic lobectomy: short-term results of a randomized clinical trial (RVlob trial). Ann Surg. (2022) 275:295–302. doi: 10.1097/SLA.0000000000004922, PMID: 33938492

[11] Kent MS Hartwig MG Vallieres E Abbas AE Cerfolio RJ Dylewski MR. Pulmonary open, robotic, and thoracoscopic lobectomy (PORTaL) study: an analysis of 5721 cases. Ann Surg. (2023) 277:528–33. doi: 10.1097/SLA.0000000000005115, PMID: 34534988 PMC9891268

[12] Schwartz G Sancheti M Blasberg J. Robotic thoracic surgery. Surg Clin North Am. (2020) 100:237–48. doi: 10.1016/j.suc.2019.12.001, PMID: 32169178

[13] Diao H Xu L Li X Wang Y Peng Z. Comparison results of three-port robot-assisted and uniportal video-assisted lobectomy for functional recovery index in the treatment of early stage non-small cell lung cancer: A propensity score-matched analysis. Ann Surg Oncol. (2024) 31:2470–81. doi: 10.1245/s10434-023-14767-8, PMID: 38105381 PMC10908624

[14] Wang YC Diao HX Xu L Peng ZM. Comparison of early functional recovery following triportal robot-assisted and uniportal video-assisted segmentectomy in patients with early-stage non-small cell lung cancer: A propensity score-matched analysis. Thorac Cancer. (2025) 16:e70041. doi: 10.1111/1759-7714.70041, PMID: 40074689 PMC11903195

[15] Haruki T Kubouchi Y Kidokoro Y Matsui S Ohno T Kojima S. A comparative study of robot-assisted thoracoscopic surgery and conventional approaches for short-term outcomes of anatomical segmentectomy. Gen Thorac Cardiovasc Surg. (2024) 72:338–45. doi: 10.1007/s11748-023-01983-y, PMID: 37934374 PMC11018688

[16] Wang Y Meng C Shi L Gu S Fan X Wang Q. Short-term outcomes of robotic- vs. television-assisted thoracoscopic segmental lung resection for early-stage non-small-cell lung cancer in the day surgery models. J Thorac Dis. (2024) 16:7257–70. doi: 10.21037/jtd-24-1020, PMID: 39678901 PMC11635280

[17] Haruki T Yamamoto H Hoshikawa Y Iwata H Sato Y Suzuki K. Clinicopathological features and perioperative outcomes of robot-assisted thoracoscopic surgery for primary lung cancer: An analysis of initial outcomes based on the National Clinical Database. Surg Today. (2025). doi: 10.1007/s00595-025-02992-5, PMID: 39918571

[18] Liang H Liang W Zhao L Chen D Zhang J Zhang Y. Robotic versus video-assisted lobectomy/segmentectomy for lung cancer: A meta-analysis. Ann Surg. (2018) 268:254–9. doi: 10.1097/SLA.0000000000002346, PMID: 28628562

[19] Zhou Q Huang J Pan F Li J Liu Y Hou Y. Operative outcomes and long-term survival of robotic-assisted segmentectomy for stage IA lung cancer compared with video-assisted thoracoscopic segmentectomy. Transl Lung Cancer Res. (2020) 9:306–15. doi: 10.21037/tlcr-20-533, PMID: 32420070 PMC7225141

[20] Veronesi G. Robotic thoracic surgery: technical considerations and learning curve for pulmonary resection. Thorac Surg Clin. (2014) 24:135–41. doi: 10.1016/j.thorsurg.2014.02.009, PMID: 24780416

[21] Yang F Chen L Wang H Wang Q Yang C. Optimizing surgical precision: a comparative study of three-port vs. four-port robotic-assisted lobectomy for NSCLC. J Cardiothorac Surg. (2025) 20:184. doi: 10.1186/s13019-025-03436-4, PMID: 40217318 PMC11987412

[22] Rajaram R Mohanty S Bentrem DJ Pavey ES Odell DD Bharat A. Nationwide assessment of robotic lobectomy for non-small cell lung cancer. Ann Thorac Surg. (2017) 103:1092–100. doi: 10.1016/j.athoracsur.2016.09.108, PMID: 28109575 PMC6714562

[23] Mitzman B Smith BK Varghese TJ. Resident training in robotic thoracic surgery. Thorac Surg Clin. (2023) 33:25–32. doi: 10.1016/j.thorsurg.2022.07.009, PMID: 36372530

[24] Wilson-Smith AR Anning N Muston B Eranki A Williams ML Wilson-Smith CJ. The learning curve of the robotic-assisted lobectomy-a systematic review and meta-analysis. Ann Cardiothorac Surg. (2023) 12:1–8. doi: 10.21037/acs-2022-urats-14, PMID: 36793987 PMC9922770

[25] Merritt RE Kneuertz PJ D’Souza DM. Successful transition to robotic-assisted lobectomy with previous proficiency in thoracoscopic lobectomy. Innov (Phila). (2019) 14:263–71. doi: 10.1177/1556984519845672, PMID: 31050320

[26] Kneuertz PJ Cheufou DH D’Souza DM Mardanzai K Abdel-Rasoul M Theegarten D. Propensity-score adjusted comparison of pathologic nodal upstaging by robotic, video-assisted thoracoscopic, and open lobectomy for non-small cell lung cancer. J Thorac Cardiovasc Surg. (2019) 158:1457–66. doi: 10.1016/j.jtcvs.2019.06.113, PMID: 31623811

[27] Kwon ST Zhao L Reddy RM Chang AC Orringer MB Brummett CM. Evaluation of acute and chronic pain outcomes after robotic, video-assisted thoracoscopic surgery, or open anatomic pulmonary resection. J Thorac Cardiovasc Surg. (2017) 154:652–9. doi: 10.1016/j.jtcvs.2017.02.008, PMID: 28291605

[28] Sawabata N Maeda H Takeda S Inoue M Koma M Tokunaga T. Persistent cough following pulmonary resection: observational and empiric study of possible causes. Ann Thorac Surg. (2005) 79:289–93. doi: 10.1016/j.athoracsur.2004.06.045, PMID: 15620960

[29] Novellis P Maisonneuve P Dieci E Voulaz E Bottoni E Di Stefano S. Quality of life, postoperative pain, and lymph node dissection in a robotic approach compared to VATS and OPEN for early stage lung cancer. J Clin Med. (2021) 10:1687. doi: 10.3390/jcm10081687, PMID: 33920023 PMC8071041

[30] Yang X Yin D Chen SQ. Effect of nursing on postoperative respiratory function and mental health of lung cancer patients. World J Clin cases. (2024) 12:922–30. doi: 10.12998/wjcc.v12.i5.922, PMID: 38414608 PMC10895621

[31] Rajaram R Rice DC Li Y Bruera E Liu E Song C. Postoperative opioid use after lobectomy for primary lung cancer: A propensity-matched analysis of Premier hospital data. J Thorac Cardiovasc Surg. (2021) 162:259–68. doi: 10.1016/j.jtcvs.2020.04.148, PMID: 32718706

[32] Nasir BS Bryant AS Minnich DJ Wei B Cerfolio RJ. Performing robotic lobectomy and segmentectomy: cost, profitability, and outcomes. Ann Thorac Surg. (2014) 98:203–208, 208-209. doi: 10.1016/j.athoracsur.2014.02.051, PMID: 24793685

[33] Kneuertz PJ Singer E D’Souza DM Abdel-Rasoul M Moffatt-Bruce SD Merritt RE. Hospital cost and clinical effectiveness of robotic-assisted versus video-assisted thoracoscopic and open lobectomy: A propensity score-weighted comparison. J Thorac Cardiovasc Surg. (2019) 157:2018–26. doi: 10.1016/j.jtcvs.2018.12.101, PMID: 30819575

[34] Servais EL Smit PJ. Robotic thoracic surgery: current practices and emerging technologies. Thorac Surg Clin. (2023) 33:xiii–xiv. doi: 10.1016/j.thorsurg.2022.09.001, PMID: 36372540

[35] Kneuertz PJ D’Souza DM Richardson M Abdel-Rasoul M Moffatt-Bruce SD Merritt RE. Long-term oncologic outcomes after robotic lobectomy for early-stage non-small-cell lung cancer versus video-assisted thoracoscopic and open thoracotomy approach. Clin Lung Cancer. (2020) 21:214–24. doi: 10.1016/j.cllc.2019.10.004, PMID: 31685354

